# Impact of omalizumab on treatment of severe allergic asthma in UK clinical practice: a UK multicentre observational study (the APEX II study)

**DOI:** 10.1136/bmjopen-2016-011857

**Published:** 2016-08-09

**Authors:** Robert M Niven, Dinesh Saralaya, Rekha Chaudhuri, Matthew Masoli, Ian Clifton, Adel H Mansur, Victoria Hacking, Susan McLain-Smith, Andrew Menzies-Gow

**Affiliations:** 1 Manchester Academic Health Science Centre, The University of Manchester & University Hospital of South Manchester, Manchester, UK; 2 Department of Respiratory Medicine, Bradford Teaching Hospitals NHS Foundation Trust, Bradford, UK; 3 Department of Respiratory Medicine, Gartnavel General Hospital, Glasgow, UK; 4 Department of Respiratory Medicine, Plymouth Hospitals NHS Trust, Plymouth, UK; 5 Department of Respiratory Medicine, St James's University Hospital, Leeds, UK; 6 Birmingham Regional Severe Asthma Service, Birmingham Heartlands Hospital, Birmingham, UK; 7 Novartis Pharmaceuticals UK Ltd, Frimley, UK; 8 pH Associates, Marlow, UK; 9 Department of Asthma and Allergy, Royal Brompton Hospital, London, UK

**Keywords:** Severe asthma, omalizumab, corticosteroids, exacerbations

## Abstract

**Objective:**

To describe the impact of omalizumab on asthma management in patients treated as part of normal clinical practice in the UK National Health Service (NHS).

**Design:**

A non-interventional, mixed methodology study, combining retrospective and prospective data collection for 12 months pre-omalizumab and post-omalizumab initiation, respectively.

**Setting:**

Data were collected in 22 UK NHS centres, including specialist centres and district general hospitals in the UK.

**Participants:**

258 adult patients (aged ≥16 years; 65% women) with severe persistent allergic asthma treated with omalizumab were recruited, of whom 218 (84.5%) completed the study.

**Primary and secondary outcome measures:**

The primary outcome measure was change in mean daily dose of oral corticosteroids (OCS) between the 12-month pre-omalizumab and post-omalizumab initiation periods. A priori secondary outcome measures included response to treatment, changes in OCS dosing, asthma exacerbations, lung function, employment/education, patient-reported outcomes and hospital resource utilisation.

**Results:**

The response rate to omalizumab at 16 weeks was 82.4%. Comparing pre-omalizumab and post-omalizumab periods, the mean (95% CIs) daily dose of OCS decreased by 1.61 (−2.41 to −0.80) mg/patient/day (p<0.001) and hospital exacerbations decreased by 0.97 (−1.19 to −0.75) exacerbations/patient (p<0.001). Compared with baseline, lung function, assessed by percentage of forced expiratory volume in 1 s, improved by 4.5 (2.7 to 6.3)% at 16 weeks (p<0.001; maintained at 12 months) and patient quality of life (Asthma Quality of Life Questionnaire) improved by 1.38 (1.18 to 1.58) points at 16 weeks (p<0.001, maintained at 12 months). 21/162 patients with complete employment data gained employment and 6 patients lost employment in the 12-month post-omalizumab period. The mean number of A&E visits, inpatient hospitalisations, outpatient visits (excluding for omalizumab) and number of bed days/patient decreased significantly (p<0.001) in the 12-month post-omalizumab period.

**Conclusions:**

These data support the beneficial effects of omalizumab on asthma-related outcomes, quality of life and resource utilisation in unselected patients treated in ‘real-world’ clinical practice.

**Strengths and limitations of this study**

- Data for Asthma Patient Experience with Xolair (APEX) II were collected in a non-interventional clinical setting, and therefore, the results reflect the outcomes of treatment delivered according to normal UK clinical practice, using National Institute for Health and Care Excellence guidance, but in otherwise unselected patients.
- The study was limited by the necessity to collect pre-omalizumab data retrospectively, which affected the quality and availability of data, and is reflected in the volume of missing data for some of the variables, leading to differences in the number of patients for each analysis; in particular, details of OCS prescriptions provided in primary care were limited in the pre-omalizumab period, which may have led to underestimation of the number of patients classified as ‘on continuous corticosteroids (CCS)’ and calculation of the mean daily dose in the pre-omalizumab period.
- The prospective data collection in the post-omalizumab period allowed for more complete data collection related to OCS use and asthma-related outcomes than in the pre-omalizumab period; in particular, details of primary care OCS prescriptions were limited in the pre-omalizumab period; the mixed study design leads to a potential underestimation of the beneficial effects of omalizumab.
- Data related to days off sick from work/education were subject to recall bias in pre-omalizumab and post-omalizumab periods; however, the data are included due to its novelty and potential importance to the overall societal benefit of optimally treating severe asthma.

## Introduction

Asthma is a common chronic inflammatory disease of the airways, with an estimated lifetime prevalence of one in nine people in England.^1^ Asthma is difficult to control in many patients, with a recent European study reporting that 76% of patients are poorly controlled, though causes of poor control are multifactorial.^2^ IgE plays a central role in mediating inflammatory reactions associated with allergic asthma via interactions with high-affinity receptors on mast cells and basophils.^3^ Asthma exacerbations are common and associated with significant morbidity and mortality,^4^ and increased serum IgE levels to common inhaled allergens is a risk factor for asthma exacerbations.^5^ ^6^ Severe asthma is reported as occurring in 5–10% of patients and frequently requires long-term treatment with oral corticosteroids (OCS) or frequent short burst use of high-dose OCS to try and improve asthma control and prevent exacerbations, although some patients remain poorly controlled despite this treatment.^7^ ^8^ OCS usage for asthma management is associated with adverse effects, including obesity, mood disturbances, development of osteoporosis, fractures, glaucoma, cataracts, endocrine and metabolic disorders, muscle weakness and cardiovascular disorders.^8–12^ Therapeutic options that improve asthma control, reduce asthma exacerbations and reduce reliance on OCS use in patients with severe asthma are required to improve disease-related and current therapy-related morbidity.

Omalizumab (Xolair) is a recombinant humanised monoclonal antibody that binds to IgE and is indicated as add-on therapy to improve asthma control in patients with severe persistent allergic asthma.^13^ Omalizumab decreases expression of high-affinity IgE receptors on inflammatory cells and airway accumulation of eosinophils.^6^ ^14^ Omalizumab has been demonstrated in clinical trials and observational studies (including a retrospective study of patients treated with omalizumab in the UK; the Asthma Patient Experience with Xolair (APEX) I study) to significantly decrease OCS use, asthma exacerbations, Accident and Emergency (A&E) visits, hospitalisations and bed days, and to improve asthma symptoms, lung function and patients’ quality of life (QoL).^14–16^ Patients with severe allergic asthma are at increased risk for exacerbations requiring hospitalisation, which adversely affects their QoL, employment, hospital resource utilisation and may result in death.^16–18^ There are limited prospective data available describing the impact of omalizumab on the management of patients with severe asthma when used in a real-world clinical setting.

The aim of the APEX II study was to describe the impact of omalizumab on asthma management in adult patients (aged 16 years and over) being treated with omalizumab as part of normal clinical practice within the UK National Health Service (NHS). This was carried out using a prospective methodology for collection of data from the point of decision to prescribe omalizumab, to address, as far as possible, the limitations of the previous retrospective study.^16^

## Methods

### Study design and setting

APEX II involved collecting data on patients from UK NHS secondary care centres using omalizumab as part of normal clinical practice, including specialist centres and district general hospitals, from across the UK. The study employed a mixed methodology to obtain retrospective data on the 12-month period prior to omalizumab initiation (the pre-omalizumab period) and prospective data at omalizumab initiation (baseline) and during the 12-month period following omalizumab initiation (the post-omalizumab period; evaluated at 16 weeks, 8 months and 12 months following omalizumab initiation). Data were collected between 20 January 2012 and 1 February 2015.

### Patients

All eligible adult patients (aged 16 years or over) with severe persistent allergic (IgE-mediated) asthma for whom omalizumab was prescribed for the first time as part of normal clinical practice were invited to take part in the study by a member of the clinical team. Patients with insufficient baseline data and those who had undergone bronchial thermoplasty or were participating in any interventional trial of asthma treatment were excluded from the study. Patients gave written informed consent according to a protocol approved by the UK National Research Ethics Service (REC reference number 11/LO/2025). Local hospital management approval was obtained in each participating centre.

### Data collection

Retrospective data for the 12-month pre-omalizumab period for each patient were collected from paper-based and electronic medical records. Where data were incomplete in study site medical records, additional data relating to the 12-month pre-omalizumab period were requested from the primary/secondary care records held by general practitioners and referring hospitals. Prospective data were recorded by members of the clinical team (usually a Clinical Nurse Specialist) during routine clinical consultations with patients at omalizumab initiation and in the post-omalizumab period (16 weeks (±2 weeks), 8 months (±1 month) and 12 months (±1 month)). All data were collected in anonymised-coded form on standard data collection forms designed for the study. All staff participating in data collection were trained in the study documentation requirements and were required to maintain patient confidentiality and to report any adverse events to the study sponsor in accordance with the Medicines and Healthcare Products Regulatory Agency requirements.

### Outcome measures

The primary outcome measure was the change in mean daily dose of OCS prescribed per patient between the 12-month pre-omalizumab and post-omalizumab initiation periods. Secondary outcome measures included patient demographic and clinical characteristics; response to treatment; proportion of patients stopping and/or reducing OCS by ≥20% in the post-omalizumab period; changes between the 12-month pre-omalizumab and post-omalizumab initiation periods in the number of asthma exacerbations (exacerbations were defined as ‘hospital exacerbations’ when patients attended A&E or were admitted and defined as ‘dose exacerbations’ when OCS dose increased by ≥10 mg at any point for at least 3 days), NHS secondary care resource utilisation, number of working/education days lost and number of patients employed/unemployed; changes between post-omalizumab visits and omalizumab initiation visit in weight, lung function (forced expiratory volume in 1 s (FEV_1_(L)) and expressed as a percentage of predicted (FEV_1_%)) and patient-reported outcomes (PRO; using validated PRO measures: Asthma Control Test (ACT), Asthma Quality of Life Questionnaire (AQLQ) and the EuroQol five-dimensions (EQ-5D) health questionnaire).

### Statistical analyses

The retrospective APEX I study^16^ demonstrated a reduction in mean total quantity of OCS prescribed in the 12-month period post-omalizumab initiation of 1.87 g (SD of paired differences=2.7 g). A sample size of 250 patients was, therefore, considered to be sufficient to reliably describe a similar reduction at p=0.01.

APEX II was a non-interventional study and all analyses were conducted using the available data based on the intention to treat (ITT). No imputation of missing data was undertaken and the number available for each analysis is stated where data were missing. Patient demographic and clinical characteristics were summarised for all patients using descriptive statistics (contingency tables for qualitative variables) and mean (SD) or median (range) for quantitative variables. Primary and secondary outcome measures were compared using the Student's t-test (comparing 12-month pre-omalizumab and post-omalizumab initiation periods for OCS use, asthma exacerbations, employment and resource utilisation (except for the number of intensive care unit (ICU) admissions which was compared using the McNemar test) and comparing post-omalizumab visits (16 weeks, 8 months and 12 months) with omalizumab initiation visit (baseline) for lung function, PROs and weight), and reported as the mean difference with 95% CIs and p values.

Subgroup analyses were conducted on data from the subgroup of patients on continuous OCS for at least 6 months prior to initiation of omalizumab (baseline continuous corticosteroid (CCS) subgroup) and separately in the subgroup classified as responders to omalizumab at the 16-week assessment visit. As this was a real-world non-interventional study, the subgroup of responders comprised all patients who were classified by the treating physician as responders at the 16-week assessment according to usual clinical practice at each centre. Participating centres were expected to be following guidelines (based on National Institute for Health and Care Excellence (NICE) or Scottish Medicines Consortium (SMC) criteria); however, all patients for whom funding was obtained from local commissioners were included.

## Results

A total of 258 patients from 22 centres were recruited into the study. Details of the participating centres are presented in online supplementary table S1.

10.1136/bmjopen-2016-011857.supp1 Supplementary figures and tables

### Patient baseline characteristics

The baseline characteristics of the patients included in the study are presented in table 1. Of 258 patients recruited, 65.1% were women and 62.4% had never smoked (median pack-years in current/ex-smokers (where available, n=52): 10.0 (range: 0.5–80.0)). The mean age at diagnosis was 19.7 years and the mean duration of asthma was 25.1 years (table 1). The most common allergies were to house dust mite (68.2%), animal fur (66.7%) and pollen (48.1%), with eight patients (3.1%) having no documented allergies (table 1) and 226 (87.6%) patients recorded as allergic to one or more perennial aeroallergens. The most commonly reported comorbidities (see table 1) were perennial (28.7%) and seasonal (19.0%) rhinitis, nasal polyps (14.3%) and sinusitis (13.6%). The patient characteristics of the CCS subgroup (n=76) were similar to those of patients who were not on CCS (‘non-CCS’, n=136) at baseline (see online supplementary table S2; insufficient data to classify OCS use, n=46). Of the 214 patients treated with omalizumab at centres in England, 193 (90.2%) were compliant with NICE guidance and all 33 patients in Scotland (100%) were compliant with SMC guidance (not applicable for n=11 patients from Belfast). Of the original study population, 218 (84.5%) patients completed the study (see online supplementary figure S1).

**Table 1:** Baseline patient characteristics

Variable	Total patient cohort (n=258)
Age (years)	44.7 (14.2)
Female	168 (65.1%)
Smoker	
Never	161 (62.4%)
Ex	83 (32.2%)
Current	6 (2.3%)
Unknown	8 (3.1%)
Weight (kg)	82.4 (20.1)
BMI (kg/m^2^)*	29.9 (6.8)
Ethnicity	
White British	224 (86.8%)
Pakistani	19 (7.4%)
Other	15 (5.8%)
Duration of asthma (years)†	25.1 (15.1)
Age at asthma diagnosis (years)†	19.7 (17.9)
Allergies (most common)	
House dust mite	176 (68.2%)
Animal fur	172 (66.7%)
Pollen	124 (48.1%)
Mould	88 (34.1%)
Plant material	86 (33.3%)
None documented	8 (3.1%)
Reported comorbidities (selected)	
Perennial rhinitis	74 (28.7%)
Seasonal rhinitis	49 (19.0%)
Nasal polyps	37 (14.3%)
Sinusitis	35 (13.6%)
Previous anaphylaxis	22 (8.5%)
Bronchiectasis	9 (3.5%)
Diabetes	5 (1.9%)
None	74 (28.7%)

Data presented as mean (SD) or n (%).

*One patient did not have height recorded.

†Duration of asthma not recorded for 41 patients.

BMI, body mass index.

### Omalizumab dosing

Most patients (n=220; 84.9%) were dosed correctly from available weight/baseline IgE measurements. Of the remaining patients, 17 were eligible for dosing but received the incorrect dose (based on recorded weight/baseline IgE measurements), while 21 patients were treated when their IgE levels or weight exceeded the upper limits (all patients in this latter group received 600 mg every 2 weeks). The mean duration of omalizumab treatment was 304 days (SD: 104) with a mean of 14.5 (SD: 7.1) omalizumab injections per patient during the post-omalizumab study period.

### Response to omalizumab

In the overall ITT patient group, there were 239 patients with omalizumab response classified by treating physicians at the 16-week assessment; 197 patients were classified as responders and 42 as non-responders (response rate: 82.4%; indeterminate response, n=6). Patients classified as responders based on clinical assessment at 16 weeks were analysed as the responder subgroup in all subsequent analyses (the characteristics of responders and non-responders are presented in online supplementary table S2). The response rate was similar in the CCS subgroup (61/73; 83.6%). At the end of the 12-month post-omalizumab observation period, 179/258 patients (69.4%) remained on omalizumab (see online supplementary table S3 for reasons for discontinuing).

### Impact of omalizumab on OCS use

**Figure 1:**
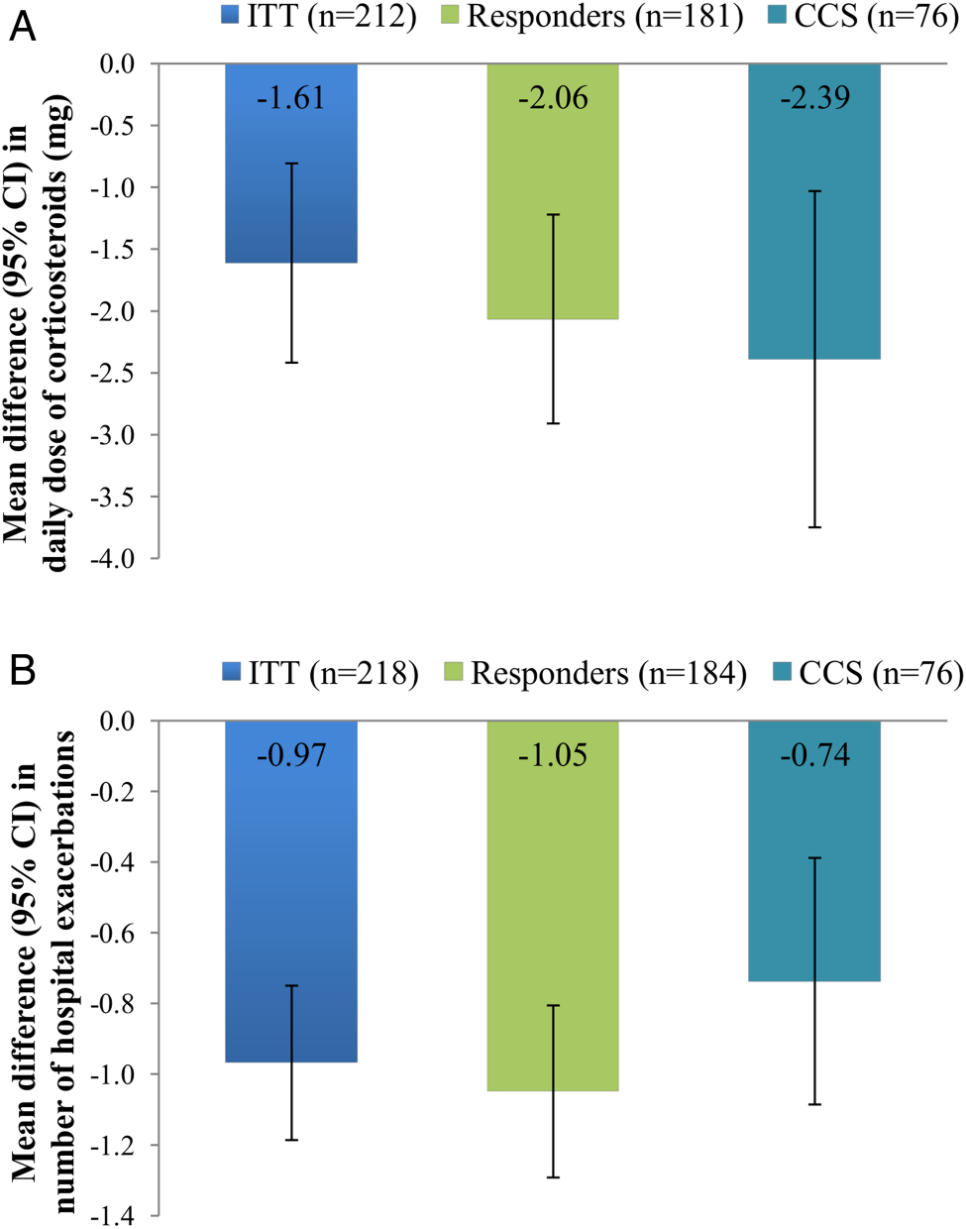
Omalizumab treatment outcomes. (A) Steroid sparing effect of omalizumab. (B) Impact of omalizumab on hospital exacerbations. Data are presented as mean difference (95% CIs) between the 12-month pre-omalizumab and post-omalizumab periods. Paired t-test, p<0.001 for each comparison. CCS, continuous corticosteroid; ITT, intention to treat.

The mean daily dose of OCS reduced significantly during the study period, as shown in figure 1A. In the ITT population, mean daily dose decreased from 10.37 mg/day per patient pre-omalizumab to 8.76 mg/day per patient post-omalizumab (p<0.001). Similar significant decreases in mean daily OCS dose were observed in the responder subgroup (9.83 mg/day per patient pre-omalizumab vs 7.77 mg/day per patient post-omalizumab, p<0.001) and the CCS subgroup (15.40 mg/day pre-omalizumab vs 13.01 mg/day post-omalizumab, p<0.001). Patient weight did not differ between the baseline and the 12-month post-omalizumab visits (mean difference +0.71 (95% CI −0.16 to 1.57) kg, n=202; p>0.1).

At the end of the 12-month post-omalizumab observation period, 15.8% (12/76) of the patients on CCS at baseline had stopped taking OCS completely, with 42.1% (32/76) of the CCS patients either stopping or reducing OCS by ≥20% at 12 months.

### Impact of omalizumab on asthma exacerbations

The mean number of ‘hospital exacerbations’ (requiring A&E attendance and/or admission) per patient decreased significantly during the study period, as shown in figure 1B. In the ITT population, mean hospital exacerbations per patient decreased from 1.66 pre-omalizumab to 0.69 post-omalizumab (p<0.001). Similar significant decreases in hospital exacerbations were observed in the responder subgroup (mean per patient 1.71 vs 0.66 pre-omalizumab and post-omalizumab, respectively, p<0.001) and the CCS subgroup (1.20 vs 0.46 pre-omalizumab and post-omalizumab, respectively, p<0.001).

Similarly, the mean number of ‘dose exacerbations’ (≥10 mg increase in OCS at any point for at least 3 days) per patient decreased significantly in the ITT population (4.58 vs 2.53 pre-omalizumab and post-omalizumab, respectively; mean difference −2.05 (95% CI −2.51 to −1.59), p<0.001). Similar decreases were observed in the responder subgroup (mean 4.62 vs 2.36 dose exacerbations pre-omalizumab and post-omalizumab; mean difference −2.26 (95% CI −2.76 to −1.76), p<0.001) and the CCS subgroup (mean 3.64 vs 2.40 dose exacerbations pre-omalizumab and post-omalizumab; mean difference −1.25 (95% CI −2.04 to −0.46), p<0.01).

### Impact of omalizumab on lung function

**Figure 2:**
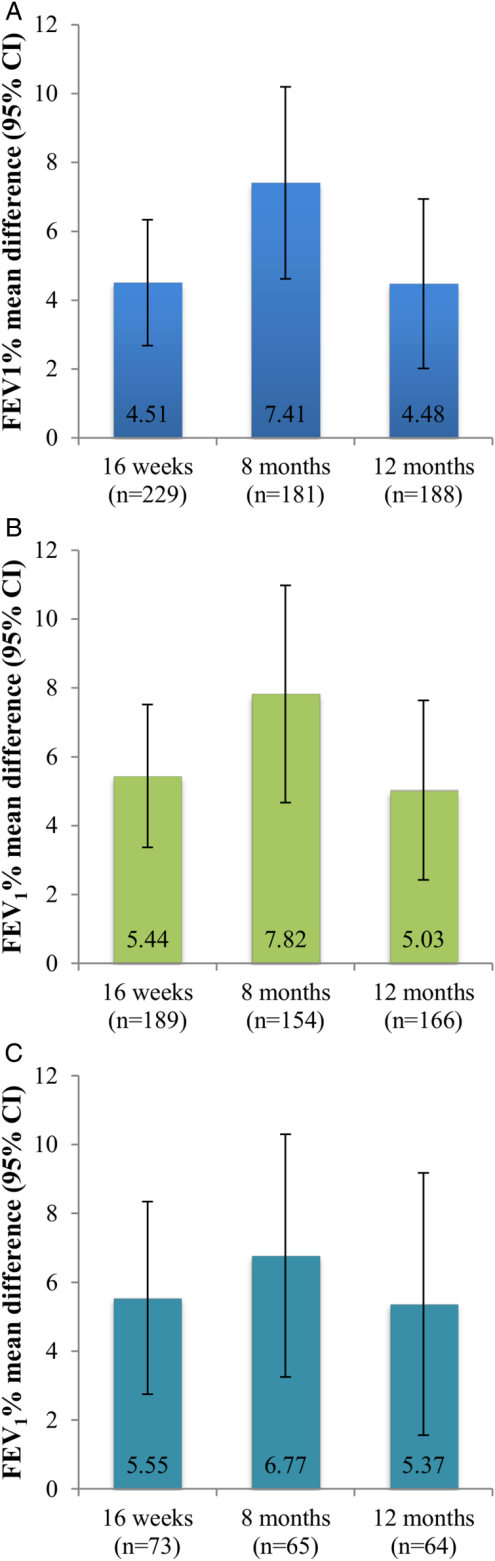
Impact of omalizumab on lung function. Data are presented as mean difference (95% CIs) comparing assessments at 16-week, 8-month and 12-month post-omalizumab with baseline. (A) ITT patients, paired t-test, p<0.001 for each comparison; (B) responder subgroup, paired t-test, p<0.001 for each comparison; (C) CCS subgroup, paired t-test, p<0.001 for 16-week and 8-month comparisons and p<0.01 for 12-month comparisons. CCS, continuous corticosteroid; FEV_1_, forced expiratory volume in 1 s; ITT, intention to treat.

Mean FEV_1_% improved significantly in the ITT group at each post-omalizumab visit—baseline: 66.8%; 16 weeks: 71.4%; 8 months: 74.6%; 12 months: 71.3%; paired t-test p<0.001 for each comparison (see figure 2A). Similar increases in FEV_1_% were observed in the responder and CCS subgroups, as shown in figure 2B, C, mirroring the FEV_1_(L) data (see online supplementary figure S2).

### Impact of omalizumab on PROs

A significant improvement in PROs was observed when assessed using asthma-specific ACT and AQLQ tools and the generic EQ-5D in the overall ITT patient group, as shown in table 2. Significant improvements in each PRO measure compared with baseline were observed at the 16-week post-omalizumab visit, which were maintained throughout the 12-month post-omalizumab observation period. Similar improvements were observed in the responder and CCS subgroups (see online supplementary tables S4 and S5).

**Table 2:** PRO measures in the ITT patient group (paired samples)

	16 weeks	8 months	12 months
ACT score	n=222	n=177	n=187
Mean (SD) at baseline	9.76 (4.29)	9.77 (4.38)	9.84 (4.34)
Mean (SD) at visit	15.01 (5.71)	14.99 (5.78)	14.41 (5.69)
Mean difference (95% CI)	5.26 (4.58 to 5.93)*	5.22 (4.43 to 6.00)*	4.57 (3.75 to 5.38)*
AQLQ score	n=192	n=149	n=161
Mean (SD) at baseline	3.27 (1.29)	3.14 (1.28)	3.20 (1.27)
Mean (SD) at visit	4.65 (1.55)	4.61 (1.54)	4.39 (1.48)
Mean difference (95% CI)	1.38 (1.18 to 1.58)*	1.47 (1.25 to 1.70)*	1.20 (0.97 to 1.42)*
EQ-5D index	n=215	n=167	n=173
Mean (SD) at baseline	0.59 (0.25)	0.58 (0.26)	0.58 (0.25)
Mean (SD) at visit	0.71 (0.25)	0.68 (0.26)	0.69 (0.26)
Mean difference (95% CI)	0.12 (0.09 to 0.15)*	0.10 (0.06 to 0.13)*	0.11 (0.08 to 0.15)*
EQ-5D VAS	n=196	n=162	n=166
Mean (SD) at baseline	54.2 (20.3)	53.5 (20.3)	54.3 (20.1)
Mean (SD) at visit	66.8 (21.2)	67.1 (21.9)	67.1 (20.9)
Mean difference (95% CI)	12.5 (9.5 to 15.5)*	13.6 (10.3 to 17.0)*	12.8 (9.4 to 16.2)*

*p<0.001.

ACT, Asthma Control Test; AQLQ, Asthma Quality of Life Questionnaire; EQ:5D VAS, EuroQol five-dimensions visual analogue scale; ITT, intention to treat; PRO, patient-reported outcome.

### Socioeconomic impact

There was a significant decrease in the mean number of days off sick from work or education following treatment (pre-omalizumab: 14.65 days; post-omalizumab 6.22 days; mean difference −8.43 (−13.51 to −3.35) days, p<0.01; n=63). There were 162 patients with complete data regarding employment status during the study period; of these, 21 of 93 (22.6%) patients who were unemployed pre-omalizumab gained employment post-omalizumab. By comparison, 6 of 69 (8.7%) patients employed pre-omalizumab became unemployed.

### Impact of omalizumab on resource utilisation

For the ITT population, the mean number of A&E visits, inpatient hospitalisations, outpatient visits (excluding visits for omalizumab administration) and number of bed days per patient decreased significantly in the 12-month post-omalizumab period compared with the 12-month pre-omalizumab period, as shown in table 3. The number of ICU admissions also decreased significantly (22 admissions for 17 patients pre-omalizumab vs 12 admissions for 7 patients post-omalizumab; McNemar test p<0.05). Similar results were observed in the responder and CCS subgroups (see online supplementary tables S6 and S7).

**Table 3:** Resource utilisation in the ITT patient group

	Pre-omalizumab (n=218)	Post-omalizumab (n=218)	Mean difference (95% CI)
A&E visits/patient	1.12 (1.71)	0.37 (0.91)	−0.75 (−0.99 to −0.52), p<0.001
Inpatient admissions/patient	1.24 (1.64)	0.56 (1.33)	−0.68 (−0.85 to −0.51), p<0.001
Outpatient visits/patient*	4.60 (2.48)	1.60 (2.07)	−3.00 (−3.43 to −2.58), p<0.001
Bed days/patient	6.61 (9.73)	3.39 (8.49)	−3.22 (−4.31 to −2.12), p<0.001
Day case visits/patient	0.03 (0.18)	0.03 (0.19)	0.00 (−0.04 to 0.03), p>0.01

Data are presented as mean (SD).

*Excluding visits for omalizumab administration.

ITT: intention to treat.

### Safety of omalizumab

A total of 43 adverse events in 24 patients suspected to be related to omalizumab treatment were reported; of these, 19 were classified as serious (see online supplementary table S8) and included one case of anaphylaxis (with subsequent discontinuation of omalizumab treatment) and one case of hypersensitivity reaction (subsequent action unknown). Two deaths were recorded (one pneumonia and one respiratory arrest), neither of which was suspected to be related to omalizumab treatment.

## Discussion

The results of the APEX II study of omalizumab used in a ‘real-world’ clinical setting in the UK confirm and add to the available data on the beneficial effects of omalizumab on asthma outcomes.^14–16^ Omalizumab treatment was associated with significant reductions in mean daily dose of OCS, asthma exacerbations, A&E visits, inpatient hospitalisations, outpatient visits (excluding those for omalizumab administration), hospital bed days and days off sick from employment/education. It was also associated with significant improvements in lung function, asthma-specific and generic PROs, and number of patients in employment.

The proportion of patients responding to omalizumab at the 16-week assessment was 82.4%, which is similar to the response rate in APEX I and other observational studies,^14–16^ ^19^ including studies using similar mixed methods approaches combining retrospective data collection pre-omalizumab initiation with prospective data collection following initiation.^20–23^ While most patients in APEX II were dosed correctly, a small proportion of patients were incorrectly dosed, including 21 patients whose IgE levels or weight exceeded the upper limit. While no information was available regarding the rationale for dosing in these patients, it is of note that 19 were classified as responders at 16 weeks and the magnitude of effect on outcomes was similar to those of patients appropriately treated according to UK guideline criteria (data not shown). The proportion of patients who were not treated according to dosing tables^13^ was lower in the present study than has been previously reported.^20^

The primary outcome measure (mean daily dose of OCS) decreased significantly in the 12-month post-omalizumab period, and 42.1% of patients on CCS at baseline had reduced or stopped OCS, consistent with the results of previous studies.^15^ ^19^ ^21^ ^23^ The magnitude of reduction in mean OCS daily dose was smaller than that observed in APEX I, although the mean daily dose pre-omalizumab was half of that observed in APEX I, which may reflect changes in NICE guidance regarding patient eligibility for omalizumab treatment in the intervening period. The differences in study design may also have contributed to the differences in the magnitude of reduction in OCS, with prospective data collection post-omalizumab initiation in APEX II resulting in a more accurate assessment of OCS use compared with APEX I. The observed reduction in OCS is indicative of improved asthma control, and more importantly, the number of patients ceasing CCS use is of considerable clinical importance given the morbidity associated with long-term OCS.^8–12^ We did not observe a decrease in weight at the 12-month visit compared with baseline despite the known association between OCS use and weight gain,^10^ suggesting that OCS-associated weight gain is not readily lost upon dose reduction and reflecting the multifactorial nature of successful weight loss.

We observed a significant reduction in asthma exacerbations (as assessed by hospitalisation (including A&E attendance) or OCS dose increase ≥10 mg for at least 3 days) in the ITT, responder and CCS subgroups. Similar reductions in exacerbations have been reported previously, although the criteria used to define exacerbations differed^16^ ^20^ ^23^ or were not specified.^21^ ^22^ We also observed a modest but significant improvement in lung function in the post-omalizumab period in all groups evaluated, which was observed by 16 weeks of treatment. While improvements in lung function compared with baseline were maintained throughout the 12-month follow-up period, similar to other studies,^16^ ^20^ the greatest improvements were observed at 8 months in the ITT, responder and CCS subgroups, which may suggest attenuation of response in some patients by 12 months.

QoL improved significantly in the ITT, responder and CCS patient groups when assessed using each of the three PRO instruments, demonstrating an improvement in asthma control (ACT score), asthma-related QoL (AQLQ) and general QoL (EQ-5D), consistent with previous studies, including one or more of these PRO measures.^16^ ^20^ ^24^ ^25^ It has been demonstrated that a change in AQLQ score of 0.5 in adults with asthma represents the minimal important difference in QoL, with changes of 1.0 and 1.5 representing moderate and large changes in QoL, respectively.^26^ The observed absolute reduction in AQLQ of 1.4 at 16 weeks in the ITT population in the present study, therefore, suggests a moderate to large effect of omalizumab on QoL. It should, however, be noted that the mean ACT scores post-omalizumab remained <19, indicating that asthma control was still classified as poor despite the observed reduction in OCS use and improvements in exacerbations and lung function. We also identified a significant reduction in time off sick from employment/education, consistent with the results of a previous small study.^22^ Importantly, we identified 21 patients who returned to work in the post-omalizumab period, suggesting a wider beneficial socioeconomic impact associated with treatment of patients with severe allergic asthma with omalizumab. While these data are subject to recall bias, the suggested improvement in work and education attendance and employment status represents an important health economic outcome and is also highly important in the context of patient QoL.

Omalizumab treatment was also associated with a significant reduction in hospital A&E visits and inpatient hospitalisations, consistent with previous studies^16^ ^20–23^ and with a significant reduction in ICU admissions. This supports the beneficial effects of omalizumab in reducing resource utilisation associated with emergency and inpatient admissions. We also identified a significant reduction in outpatient attendances excluding those for omalizumab administration; however, we cannot exclude that consultant-led outpatient attendances were reduced as a result of the increased frequency with which patients were seen by another healthcare professional for omalizumab administration. The impact of omalizumab on total hospital resource utilisation should take into account the reductions in A&E and inpatient admissions and consultant-led outpatient visits in addition to the resources associated with the administration of omalizumab for health economic evaluations.

Taken together, the available data support a beneficial effect of omalizumab on asthma outcomes. However, it cannot be excluded that the reduction in OCS use and other beneficial effects observed in this and previous studies may, at least in part, be attributable to the increased frequency with which patients are seen by healthcare professionals for omalizumab injections, which may lead to an improvement in compliance with OCS and other medications.

In addition, the differing methodology for collection of data in the pre-omalizumab/post-omalizumab initiation periods in APEX II introduced a potential information bias. The pre-omalizumab period relied on the quality and completeness of medical records, which was reflected in the volume of missing data for some variables, leading to differences in the number of patients included in each analysis. In particular, details of OCS prescriptions provided in primary care were limited in the pre-omalizumab period which may have led to under (or over)estimation of the number of patients classified as on CCS and calculation of the mean daily dose in the pre-omalizumab period. In addition, any hospital or A&E visits other than at the study centre would not have been identified. In contrast, the prospective data collection in the post-omalizumab period allowed for more complete data collection related to OCS use and asthma-related outcomes. However, there were a number of patients with missing data at different time-points and overall 40 patients lost to follow-up/withdrawn/excluded/died which may have led to an overestimation of the impact of omalizumab in the ITT analyses. Given the cyclical nature of asthma symptoms, we are also unable to exclude an influence of regression to the mean on patient outcomes.

Data related to days off sick from work/education were subject to recall bias in pre-omalizumab and post-omalizumab periods; however, the recall periods between post-omalizumab visits were shorter and more likely to be accurate compared with data recalled for the 12-month pre-omalizumab period. It seems unlikely that reported employment status pre-omalizumab and post-omalizumab was subject to recall bias in the same way, and therefore, the increase in the number of patients in employment in the post-omalizumab period tends to support the observed beneficial effect of omalizumab in reducing days off sick.

A key strength of APEX II lies in the fact that data were collected in a non-interventional clinical setting, and therefore, the results reflect the outcomes of patients when treated according to normal UK clinical practice in an unselected patient population. These data can, therefore, be considered to be generalisable to the wider UK population of adult patients (aged>16 years) with allergic asthma treated with omalizumab.

In conclusion, data from the present study indicate that omalizumab used in a ‘real-world’ clinical setting in the UK is associated with decreased OCS usage, decreased asthma exacerbations, improved lung function, improved work/education attendance and employment, and decreased A&E and inpatient resource utilisation. These data are of relevance to clinicians, payers and healthcare commissioners, adding to and extending the growing body of evidence, derived from different countries and differing study designs, supporting the beneficial effects of omalizumab on asthma-related outcomes, QoL and resource utilisation in unselected patients treated in normal clinical practice. The growing evidence of a wider socioeconomic impact of omalizumab treatment merits further exploration; the long-term funding of this and other moderate to high cost monoclonal therapies should consider the impact of all societal costs in costing models. A proportion of patients coming off benefits and contributing to the economy positively may offset some of the drug and delivery of care costs if such data are reproduced.

## References

[1] Simpson CR, Sheikh A Trends in the epidemiology of asthma in England: a national study of 333,294 patients. J R Soc Med 2010;103:98–106. 10.1258/jrsm.2009.090348 20200181 PMC3072257

[2] Price D, Dale P, Elder E Types, frequency and impact of asthma triggers on patients’ lives: a quantitative study in five European countries. J Asthma 2014;51:127–35. 10.3109/02770903.2013.846369 24050523 PMC3934435

[3] Canadian Agency for Drugs and Technologies in Health. Omalizumab treatment for adults and children with allergic asthma: a review of the clinical effectiveness, cost-effectiveness, and guidelines. Ottawa (ON): Canadian Agency for Drugs and Technologies in Health, 2015 http://www.ncbi.nlm.nih.gov/books/NBK280029/ (accessed 19 May 2015). 25834884

[4] Blakey JD, Woolnough K, Fellows J Assessing the risk of attack in the management of asthma: a review and proposal for revision of the current control-centred paradigm. Prim Care Respir J 2013;22:344–52. 10.4104/pcrj.2013.00063 23817678 PMC6442819

[5] Pollart SM, Chapman MD, Fiocco GP Epidemiology of acute asthma: IgE antibodies to common inhalant allergens as a risk factor for emergency room visits. J Allergy Clin Immunol 1989;83:875–82. 10.1016/0091-6749(89)90100-0 2715548

[6] Thomson NC, Chaudhuri R Omalizumab: clinical use for the management of asthma. Clin Med Insights Circ Respir Pulm Med 2012;6:27–40. 10.4137/CCRPM.S7793 22745565 PMC3382304

[7] Chung KF, Wenzel SE, Brozek JL International ERS/ATS guidelines on definition, evaluation and treatment of severe asthma. Eur Respir J 2014;43:343–73. 10.1183/09031936.00202013 24337046

[8] Braunstahl GJ, Canvin J, Peachey G Healthcare resource utilization in patients receiving omalizumab for allergic asthma in a real-world setting. Biol Ther 2014;4:57–67. 10.1007/s13554-014-0019-z 25371373 PMC4254868

[9] Curtis JR, Westfall AO, Allison J Population-based assessment of adverse events associated with long-term glucocorticoid use. Arthritis Rheum 2006;55:420–6. 10.1002/art.21984 16739208

[10] Manson SC, Brown RE, Cerulli A The cumulative burden of oral corticosteroid side effects and the economic implications of steroid use. Respir Med 2009;103:975–94. 10.1016/j.rmed.2009.01.003 19372037

[11] Fardet L, Flahault A, Kettaneh A Corticosteroid-induced clinical adverse events: frequency, risk factors and patient's opinion. Br J Dermatol 2007;157:142–8. 10.1111/j.1365-2133.2007.07950.x 17501951

[12] van der Goes MC, Jacobs JWG, Boers M Monitoring adverse events of low-dose glucocorticoid therapy: EULAR recommendations for clinical trials and daily practice. Ann Rheum Dis 2010;69:1913–19. 10.1136/ard.2009.124958 20693273

[13] Novartis Pharmaceuticals. Summary of product characteristics: Xolair 2014 http://www.ema.europa.eu/docs/en_GB/document_library/EPAR_-_Product_Information/human/000606/WC500057298.pdf (accessed 18 May 2015).

[14] Normansell R, Walker S, Milan SJ Omalizumab for asthma in adults and children. Cochrane Database Syst Rev 2014;CD003559 10.1002/14651858.CD003559.pub4 24414989 PMC10981784

[15] Norman G, Faria R, Paton F Omalizumab for the treatment of severe persistent allergic asthma: a systematic review and economic evaluation. Health Technol Assess 2013;17:1–342. 10.3310/hta17520 PMC4781123 24267198

[16] Barnes N, Menzies-Gow A, Mansur AH Effectiveness of omalizumab in severe allergic asthma: a retrospective UK real-world study. J Asthma 2013;50:529–36. 10.3109/02770903.2013.790419 23574000 PMC3681088

[17] Peters SP, Ferguson G, Deniz Y Uncontrolled asthma: a review of the prevalence, disease burden and options for treatment. Respir Med 2006;100:1139–51. 10.1016/j.rmed.2006.03.031 16713224

[18] Healthcare Quality Improvement Partnership, Royal College of Physicians of London. Why asthma still kills: the National review of asthma deaths (NRAD) 2014 https://www.rcplondon.ac.uk/sites/default/files/why-asthma-still-kills-full-report.pdf (accessed 23 Sep 2015).

[19] Braunstahl GJ, Chlumský J, Peachey G Reduction in oral corticosteroid use in patients receiving omalizumab for allergic asthma in the real-world setting. Allergy Asthma Clin Immunol 2013;9:47 10.1186/1710-1492-9-47 24305549 PMC3879326

[20] Brusselle G, Michils A, Louis R ‘Real-life’ effectiveness of omalizumab in patients with severe persistent allergic asthma: the PERSIST study. Respir Med 2009;103:1633–42. 10.1016/j.rmed.2009.06.014 19619998

[21] Cazzola M, Camiciottoli G, Bonavia M Italian real-life experience of omalizumab. Respir Med 2010;104:1410–6. 10.1016/j.rmed.2010.04.013 20483574

[22] Gouder C, West LM, Montefort S The real-life clinical effects of 52 weeks of omalizumab therapy for severe persistent allergic asthma. Int J Clin Pharm 2015;37:36–43. 10.1007/s11096-014-0034-7 25394832

[23] Molimard M, Mala L, Bourdeix I Observational study in severe asthmatic patients after discontinuation of omalizumab for good asthma control. Respir Med 2014;108:571–6. 10.1016/j.rmed.2014.02.003 24565601

[24] Rubin AS, Souza-Machado A, Andradre-Lima M Effect of omalizumab as add-on therapy on asthma-related quality of life in severe allergic asthma: a Brazilian study (QUALITX). J Asthma 2012;49:288–93. 10.3109/02770903.2012.660297 22356355

[25] Korn S, Thielen A, Seyfried S Omalizumab in patients with severe persistent allergic asthma in a real-life setting in Germany. Respir Med 2009;103:1725–31. 10.1016/j.rmed.2009.05.002 19515548

[26] Juniper EF, Guyatt GH, Willan A Determining a minimal important change in a disease-specific quality of life questionnaire. J Clin Epidemiol 1994;47:81–7. 10.1016/0895-4356(94)90036-1 8283197

