# Supplementary material for: Impact of omalizumab on treatment of severe allergic asthma in UK clinical practice: a UK multicentre observational study (the APEX II study)
Source: BMJ Open. 2016 Aug 9;6(8):e011857. doi: 10.1136/bmjopen-2016-011857 (PMC4985870; doi:10.1136/bmjopen-2016-011857)
Supplement: Supplementary figures and tables [file bmjopen-2016-011857supp.pdf]

**Table S1**. APEX II Principal Investigators and participating centres.

| Principal Investigator | Centre[†] | City |
|---|---|---|
| Dr A Menzies-Gow (co-Chief Investigator) | Royal Brompton Hospital | London |
| Dr R Niven (co-Chief Investigator) | Wythenshawe Hospital | Manchester |
| Dr D Saralaya/ Dr A Aziz | Bradford Royal Infirmary | Bradford |
| Dr R Chaudhuri | Gartnavel General Hospital | Glasgow |
| Dr M Masoli | Derriford Hospital | Plymouth |
| Dr C Bucknall | Stobhill ACH Hospital | Glasgow |
| Dr I Clifton | St. James's University Hospital | Leeds |
| Dr A Mansur | Birmingham Heartlands Hospital | Birmingham |
| Prof. N Barnes | The London Chest Hospital | London |
| Prof. L Heaney | Belfast City Hospital | Belfast |
| Dr R Kurukulaaratchy | Southampton General Hospital | Southampton |
| Prof. C Corrigan | Guys Hospital | London |
| Dr J Morjaria | Castle Hill Hospital | Hull |
| Dr A Hart-Thomas/ Dr A Graham | Huddersfield Royal Infirmary | Huddersfield |
| Dr S Hand | Prince Charles General Hospital | Merthyr Tydfil |
| Dr R Allcock | Queen Elizabeth II | Gateshead |
| Dr L Dobson | Torbay Hospital | Torquay |
| Dr R Gore | Lister Hospital | Stevenage |
| Dr N Segal | North Manchester General Hospital | Manchester |
| Dr S Ramamurthy | James Cook University Hospital | Middlesbrough |
| Dr M Nordstrum | St. Peter's Hospital | Chertsey |
| Dr G Davies | Singleton Hospital | Swansea |

[†]10 centres included in the original APEX I study participated in APEX II. The additional centres were selected based on those providing secondary or specialist asthma management services that used omalizumab as part of normal clinical practice, that were interested in participating in the study and contributed to a geographical spread of centres throughout the UK to provide a representative study population

**Table S2**: Baseline patient characteristics

| Variable | Responders (n=197) | Non-responders (n=42) | CCS patients (n=76) | Non-CCS patients (n=136) |
|---|---|---|---|---|
| **Age** (years) | 44.0 (14.0) | 46.5 (14.6) | 46.5 (14.0) | 43.0 (13.7) |
| **Female** | 130 (66.0%) | 28 (66.7%) | 45 (59.2%) | 94 (69.1) |
| **Smoker** | | | | |
| Never | 123 (62.4%) | 30 (71.4%) | 42 (55.3%) | 90 (66.2%) |
| Ex | 67 (34.0%) | 8 (19.0%) | 28 (36.8%) | 41 (30.1%) |
| Current | 3 (1.5%) | 3 (7.1%) | 3 (3.9%) | 2 (1.5%) |
| Unknown | 4 (2.0%) | 1 (2.4%) | 3 (3.9%) | 3 (2.2%) |
| **Weight** (kg) | 82.6 (19.9) | 81.8 (21.1) | 83.6 (21.0) | 81.9 (18.9) |
| **BMI** (kg/m$^2$) | 30.0 (6.5)† | 30.0 (8.0) | 30.1 (7.3) | 29.9 (6.3) |
| **Ethnicity** | | | | |
| White British | 167 (84.8%) | 39 (92.9%) | 70 (92.1%) | 112 (82.4%) |
| Pakistani | 16 (8.1%) | 2 (4.8%) | 3 (3.9%) | 12 (8.8%) |
| Other | 14 (7.1%) | 1 (2.4%) | 3 (3.9%) | 12 (8.8%) |
| **Duration of asthma** (years)‡ | 24.5 (14.6) | 28.9 (16.7) | 24.6 (15.3) | 26.0 (14.8) |
| **Age at asthma diagnosis** (years)‡ | 19.7 (17.4) | 18.8 (20.4) | 23.2 (20.0) | 17.2 (15.7) |
| **Allergies** | | | | |
| House dust mite | 134 (68.0%) | 32 (76.2%) | 54 (71.1%) | 92 (67.6%) |
| Animal fur | 129 (65.5%) | 30 (71.4%) | 46 (60.5%) | 96 (70.6%) |
| Pollen | 93 (47.2%) | 24 (57.1%) | 35 (46.1%) | 68 (50.0%) |
| Mould | 67 (34.0%) | 15 (35.7%) | 29 (38.2%) | 44 (32.4%) |
| Plant material | 62 (31.5%) | 17 (40.5%) | 27 (35.5%) | 43 (31.6%) |
| None documented | 6 (3.0%) | 1 (2.4%) | 2 (2.6%) | 3 (2.2%) |
| **Co-morbidities** | | | | |
| Perennial rhinitis | 61 (31.0%) | 11 (26.2%)) | 22 (28.9%)) | 44 (32.4%)) |
| Seasonal rhinitis | 42 (21.3%) | 5 (11.9%) | 10 (13.2%) | 34 (25.0%) |
| Nasal polyps | 29 (14.7%) | 5 (11.9%) | 13 (17.1%) | 18 (13.2%) |
| Sinusitis | 28 (14.2%) | 5 (11.9%) | 9 (11.8%) | 18 (13.2%) |
| Anaphylaxis | 18 (9.1%) | 4 (9.5%) | 7 (9.2%) | 12 (8.8%) |
| Bronchiectasis | 7 (3.6%) | 1 (2.4%) | 0 (0.0%) | 7 (5.1%) |
| Diabetes | 4 (2.0%) | 0 (0.0%) | 4 (5.3%) | 1 (0.7%) |
| None | 55 (27.9%) | 14 (33.3%) | 21 (27.6%) | 35 (25.7%) |

Data presented as mean (SD) or n (%); CCS: continuous corticosteroid
†One patient did not have height recorded
‡Duration of asthma not recorded for: 28 responders, 8 non-responders, 14 CCS, 18 non-CCS

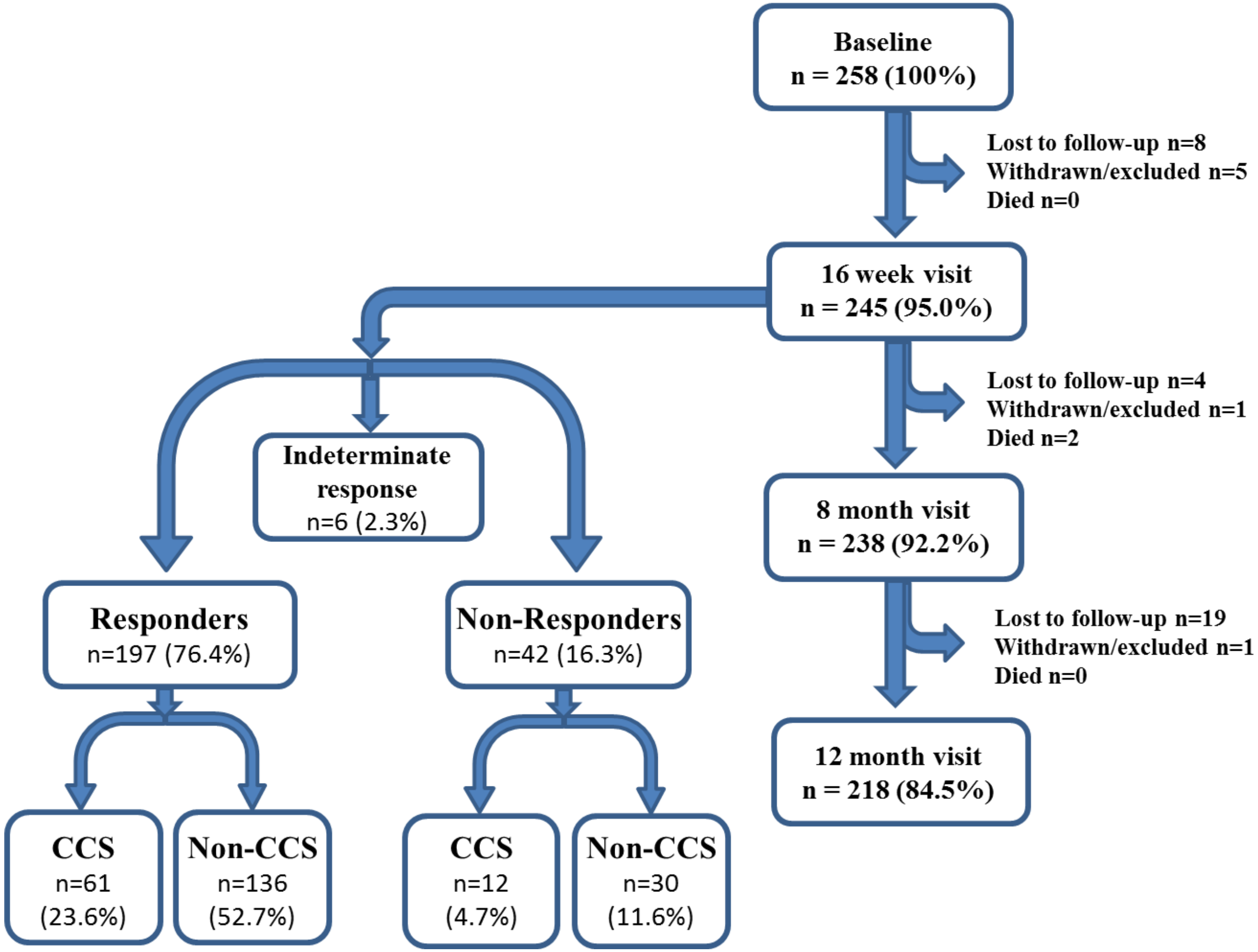


**Figure S1. APEX II study participants.**
Of the 258 patients recruited, 218 completed the 12 month visit following initiation of omalizumab. Response to omalizumab was assessed at the 16 week visit, with 197 patients being classified as responders at 16 weeks. Neither of the 2 deaths recorded was suspected to be related to omalizumab treatment. CCS: continuous corticosteroids.

**Table S3. Reasons for discontinuing omalizumab**

| Reasons for discontinuing | All patients | |
|---|---|---|
| | **n** | **%** |
| **Continuing at 12-month visit** | 179 | 69.4% |
| **Discontinuing** | 67 | 26.0% |
| **Excluded/withdrawn/died** | 9 | 3.5% |
| **Not known** | 3 | 1.2% |
| **Reasons for discontinuing:** | | |
| Lack of response | 44 | 65.7% |
| Adverse event | 4 | 6.0% |
| Patient choice | 2 | 3.0% |
| Awaiting funding | 1 | 1.5% |
| Non-compliant | 2 | 3.0% |
| Lost to follow-up | 14 | 20.9% |

**Figure S2. Impact of omalizumab on lung function: FEV1(L).** Data presented as mean difference (95% confidence intervals [CI]) comparing assessments at 16 weeks, 8 months and 12 months post-omalizumab with baseline. Panel A: Intention to treat patients, paired t-test p<0.001 for each comparison; panel B: responder subgroup, paired t-test p<0.001 for each comparison; panel C: continuous corticosteroid subgroup, paired t-test p<0.001 for 16 weeks and 8 months comparisons and p<0.01 for 12 month comparisons.

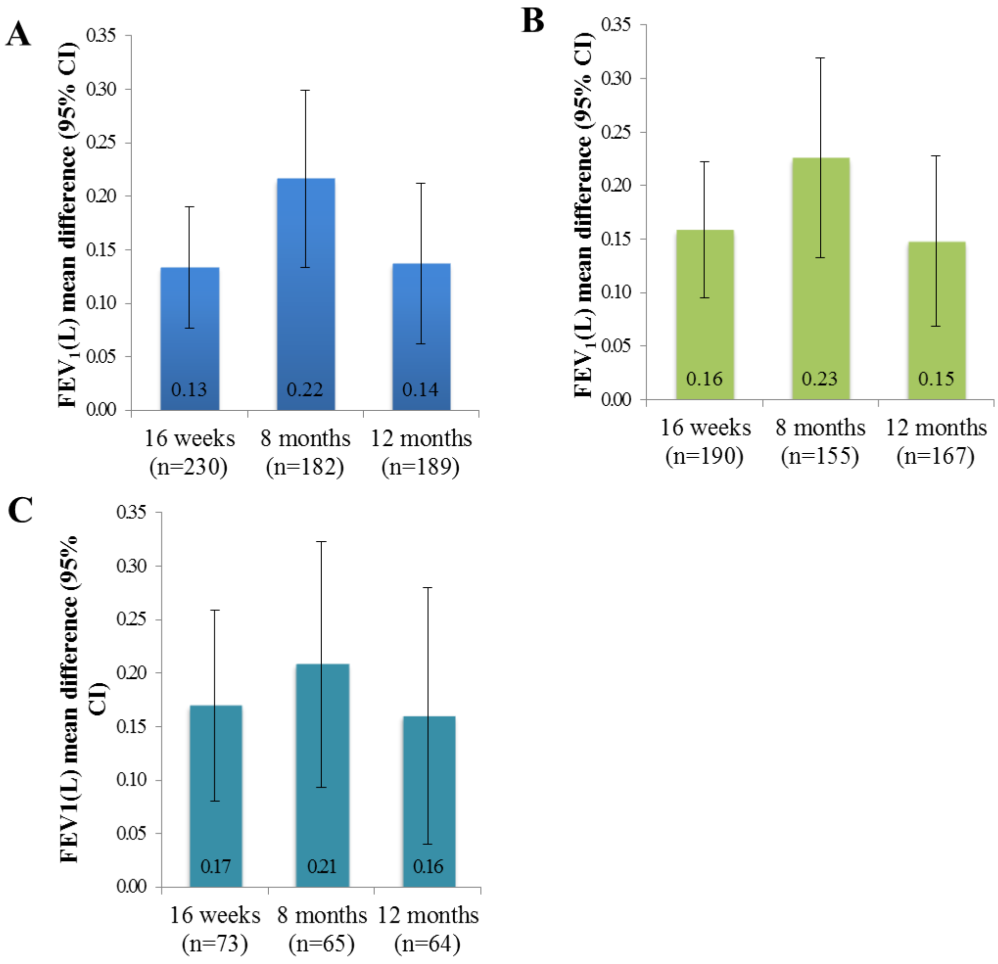

**Table S4. Patient reported outcome measures in the responder subgroup of patients**

| | Post-omalizumab initiation visit | | |
|---|---|---|---|
| | **16 weeks** | **8 months** | **12 months** |
| **ACT score** | n=179 | n=153 | n=162 |
| Mean (SD) at baseline | 9.85 (4.24) | 9.79 (4.34) | 9.93 (4.37) |
| Mean (SD) at visit | 16.18 (5.35) | 15.53 (5.68) | 15.04 (5.63) |
| Mean difference (95% CI) | 6.34 (5.60, 7.07)$^{\dagger}$ | 5.74 (4.90, 6.58)$^{\dagger}$ | 5.11 (4.23, 5.99)$^{\dagger}$ |
| **AQLQ score** | n=157 | n=130 | n=137 |
| Mean (SD) at baseline | 3.21 (1.26) | 3.12 (1.28) | 3.15 (1.26) |
| Mean (SD) at visit | 4.85 (1.50) | 4.76 (1.51) | 4.54 (1.50) |
| Mean difference (95% CI) | 1.65 (1.43, 1.86)$^{\dagger}$ | 1.64 (1.41, 1.87)$^{\dagger}$ | 1.40 (1.16, 1.64)$^{\dagger}$ |
| **EQ-5D index** | n=173 | n=146 | n=146 |
| Mean (SD) at baseline | 0.59 (0.25) | 0.58 (0.26) | 0.58 (0.24) |
| Mean (SD) at visit | 0.74 (0.24) | 0.70 (0.24) | 0.71 (0.25) |
| Mean difference (95% CI) | 0.15 (0.11, 0.18)$^{\dagger}$ | 0.12 (0.09, 0.16)$^{\dagger}$ | 0.13 (0.09, 0.16)$^{\dagger}$ |
| **EQ-5D VAS** | n=160 | n=142 | n=140 |
| Mean (SD) at baseline | 54.6 (20.6) | 53.7 (20.1) | 54.1 (20.4) |
| Mean (SD) at visit | 69.7 (20.4) | 68.6 (21.6) | 69.2 (19.9) |
| Mean difference (95% CI) | 15.1 (11.9, 18.4)$^{\dagger}$ | 14.9 (11.3, 18.5)$^{\dagger}$ | 15.0 (11.3, 18.7)$^{\dagger}$ |

$^{\dagger}p<0.001$
ACT: Asthma Control Test; AQLQ: Asthma Quality of Life Questionnaire; EQ-5D VAS: EQ-5D visual analogue scale

**Table S5. Patient reported outcome measures in the continuous corticosteroid subgroup of patients**

| | Post-omalizumab initiation visit | | |
|---|---|---|---|
| | **16 weeks** | **8 months** | **12 months** |
| **ACT score** | n=66 | n=62 | n=63 |
| Mean (SD) at baseline | 9.65 (4.46) | 9.84 (4.85) | 9.75 (4.66) |
| Mean (SD) at visit | 14.18 (5.67) | 14.00 (5.98) | 13.59 (5.66) |
| Mean difference (95% CI) | 4.53 (3.27, 5.79)$^{\dagger}$ | 4.16 (2.97, 5.36)$^{\dagger}$ | 3.84 (2.48, 5.21)$^{\dagger}$ |
| **AQLQ score** | n=57 | n=53 | n=54 |
| Mean (SD) at baseline | 3.28 (1.41) | 3.20 (1.40) | 3.20 (1.34) |
| Mean (SD) at visit | 4.46 (1.59) | 4.41 (1.59) | 4.29 (1.52) |
| Mean difference (95% CI) | 1.19 (0.76, 1.61)$^{\dagger}$ | 1.21 (0.81, 1.60)$^{\dagger}$ | 1.09 (0.69, 1.48)$^{\dagger}$ |
| **EQ-5D index** | n=66 | n=60 | n=59 |
| Mean (SD) at baseline | 0.56 (0.25) | 0.55 (0.26) | 0.56 (0.26) |
| Mean (SD) at visit | 0.69 (0.24) | 0.65 (0.25) | 0.68 (0.27) |
| Mean difference (95% CI) | 0.13 (0.08, 0.19)$^{\dagger}$ | 0.10 (0.05, 0.15)$^{\dagger}$ | 0.12 (0.06, 0.18)$^{\dagger}$ |
| **EQ-5D VAS** | n=60 | n=57 | n=57 |
| Mean (SD) at baseline | 56.6 (20.3) | 54.5 (19.4) | 58.5 (20.2) |
| Mean (SD) at visit | 64.7 (21.6) | 65.2 (20.9) | 67.7 (20.9) |
| Mean difference (95% CI) | 8.1 (2.1, 14.0)$^{\ddagger}$ | 10.7 (5.5, 15.8)$^{\dagger}$ | 9.2 (3.0, 15.4)$^{\ddagger}$ |

$^{\dagger}p<0.001$; $^{\ddagger}p<0.01$

ACT: Asthma Control Test; AQLQ: Asthma Quality of Life Questionnaire; EQ-5D VAS: EQ-5D visual analogue scale

**Table S6. Resource utilisation in the responder subgroup**

| | **Pre-omalizumab** (n=184) | **Post-omalizumab** (n=184) | **Mean difference** (95% CI) |
|---|---|---|---|
| **A&E visits/patient** | 1.19 (1.80) | 0.31 (0.84) | -0.88 (-1.14, -0.62) p<0.001 |
| **Inpatient admissions/patient** | 1.24 (1.70) | 0.53 (1.36) | -0.71 (-0.89, -0.52) p<0.001 |
| **Outpatient visits/patient**† | 4.62 (2.55) | 1.31 (1.94) | -3.31 (-3.77, -2.85) p<0.001 |
| **Bed days/per patient** | 6.35 (9.47) | 3.21 (8.65) | -3.14 (-4.26, -2.02) p<0.001 |
| **Day case visits/patient** | 0.03 (0.18) | 0.03 (0.21) | 0.00 (-0.04, 0.04) p>0.1 |

Data presented as mean (SD); †excluding visits for omalizumab administration

**Table S7. Resource utilisation in the continuous corticosteroid subgroup**

| | **Pre-omalizumab** (n=76) | **Post-omalizumab** (n=76) | **Mean difference** (95% CI) |
|---|---|---|---|
| **A&E visits/patient** | 0.92 (1.70) | 0.21 (0.60) | -0.71 (-1.10, -0.32) p<0.001 |
| **Inpatient admissions/patient** | 0.88 (1.52) | 0.46 (1.48) | -0.42 (-0.68, -0.16) p<0.01 |
| **Outpatient visits/patient**† | 4.33 (2.08) | 1.55 (1.88) | -2.79 (-3.42, -2.16) p<0.001 |
| **Bed days/per patient** | 4.13 (7.04) | 2.42 (7.76) | -1.71 (-3.32, -0.10) p<0.05 |
| **Day case visits/patient** | 0.01 (0.11) | 0.01 (0.11) | 0.00 (-0.04, 0.04) p>0.1 |

Data presented as mean (SD); †excluding visits for omalizumab administration

**Table S8. Serious suspected-related adverse events reported**

| **Adverse event** | **Number of Events** | **Events leading to discontinuation**[†] |
|---|---|---|
| Accident (no other information available) | 1 | |
| Anaphylactic reaction | 1 | 1 |
| Arthralgia | 2 | 1 |
| Asthma | 2 | |
| Hypersensitivity | 1 | |
| Hypophagia | 1 | 1 |
| Malaise | 1 | |
| Nausea | 1 | 1 |
| Paraesthesia oral | 2 | 1 |
| Pruritus | 1 | 1 |
| Pruritus generalised | 1 | |
| Swollen tongue | 1 | |
| Urinary tract infection | 1 | |
| Urticaria | 1 | 1 |
| Vomiting | 1 | 1 |
| Wheezing | 1 | 1 |
| **Total** | **19** | **9** |

[†]Events not mutually exclusive
